# Large Scale Synthesis of NiCo Layered Double Hydroxides for Superior Asymmetric Electrochemical Capacitor

**DOI:** 10.1038/srep18737

**Published:** 2016-01-12

**Authors:** Ruchun Li, Zhaoxia Hu, Xiaofeng Shao, Pengpeng Cheng, Shoushou Li, Wendan Yu, Worong Lin, Dingsheng Yuan

**Affiliations:** 1 Department of Chemistry, Jinan University, Guangzhou 510632, PR China

## Abstract

We report a new environmentally-friendly synthetic strategy for large-scale preparation of 16 nm-ultrathin NiCo based layered double hydroxides (LDH). The Ni_50_Co_50_-LDH electrode exhibited excellent specific capacitance of 1537 F g^−1^ at 0.5 A g^−1^ and 1181 F g^−1^ even at current density as high as 10 A g^−1^, which 50% cobalt doped enhances the electrical conductivity and porous and ultrathin structure is helpful with electrolyte diffusion to improve the material utilization. An asymmetric ultracapacitor was assembled with the N-doped graphitic ordered mesoporous carbon as negative electrode and the NiCo LDH as positive electrode. The device achieves a high energy density of 33.7 Wh kg^−1^ (at power density of 551 W kg^−1^) with a 1.5 V operating voltage.

Ultracapacitors (UCs) are attractive energy storage devices, due to their high power density and excellent cycling stability^1^,^2^,^3^,^4^. They have been widely used in electrical vehicles and microelectronic devices. Carbon based materials such as graphene^5^,^6^,^7^, carbon nanotubes^8^ and activated carbon^9^ are the most commonly used electrodes for electrochemical double layer (EDL) ultracapacitors. However, relatively low specific capacitance (~200 F g^−1^) is the major drawback for EDL capacitors. Alternatively, metal oxide^10^,^11^,^12^,^13^,^14^,^15^, metal hydroxide^16^,^17^, layered double hydroxides^5^,^18^,^19^ and conducting polymers^12^,^20^,^21^,^22^,^23^ are commonly used as pseudocapacitive materials. They store charges via superficial Faradic reactions and exhibit higher specific capacitance than EDL materials.

Among these pseudocapacitive materials, NiO and Ni(OH)_2_ have attracted a lot of attention due to their high theoretical capacitance, excellent chemical stability, low cost and low toxicity. For example, *β*-Ni(OH)_2_ achieved its theoretical value of specific capacitance up to 2358 F g^−1^ at a voltage of 0.44 V^17^. However, the relatively poor electrical conductivity (0.01~0.32 S m^−1^)^24^ of Ni(OH)_2_ or NiO is the major drawback as the electrode material. Thus, cobalt was introduced in Ni(OH)_2_ or NiO to improve the conductivity of electrode materials^25^ and raise the oxygen overpotential helpful with widening potential window^18^. It has been reported that Co^2+^ can be oxidized to conductive CoOOH during discharge process, resulting in the increase of conductivity of electrode materials^26^. Besides, the slow kinetics of Faradic reactions of NiCo based materials is another factor that limits their electrochemical performance^27^. It is desirable to develop NiCo based electrodes with large ion accessible surface area. Previous studies have primarily been focused on development of NiCo based nanomaterials with controlled morphology and enhanced surface area, such as nanorods^3^, nanowires^28^, and nanosheets^29^. However, the large scale synthesis of these nanostructures is rarely reported, while it is critical for practical applications.

Here we developed a large-scale and environmentally-friendly strategy to prepare ultrathin 2-dimensional (2D) porous Ni(OH)_2_-Co(OH)_2_ layered double hydroxide. To the best of our knowledge, this is the first report on the large-scale production of NiCo based LDH in a homogeneous ethylene glycol-water system. In comparison to the traditional oil/water methods, which use toxic or flammable solvents such as 1-butanol, toluene, formamide, dimethyl formamide (DMF) and dimethyl sulfoxide (DMSO)^30^, our approach involving non-toxic ethylene glycol-water as the solvent system is more environmentally friendly. Moreover, the as prepared porous, ultrathin LDH nature provides extremely large ion-accessible surface area, which improves the kinetics of superficial Faradic reaction. Asymmetric ultracapacitors using Ni_50_Co_50_-LDH electrode with a high mass loading of 8 mg as cathode and N-doped graphitic ordered mesoporous carbon (GOMC) as anode showed excellent performance in charge storage.

## Results

**Figure 1:**
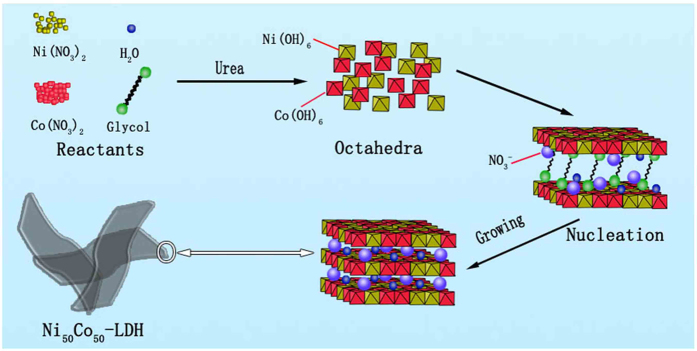
Schematic illustration of growth mechanism of Ni_50_Co_50_-LDH.

A new synthetic method was used to prepare NiCo-LDHs and Ni(OH)_2_ and Co(OH)_2_ in a homogeneous ethylene glycol-water system. The ultrathin 2D nanostructure was obtained for these materials. Among them, Ni_50_Co_50_-LDH exhibited excellent electrochemical performance, being listed in Table S1 (Support Information, SI). Therefore, Ni_50_Co_50_-LDH was mainly characterized the structure and perform the electrochemical measurement. The synthetic mechanism was illustrated in Fig. 1. Ammonia was gradually generated by the hydrolysis of urea (Eq. 1). Then NH_3_ molecules reacted with Ni and Co metal ions and formed complexes (Eq. 2). Excess amount of NH_3_ molecules produced OH^−^ and the nickel and cobalt ions were formed Ni(OH)_6_ and Co(OH)_6_ octahedra with OH^−^ (Eq. 3 and 4).


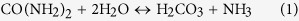



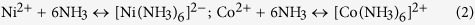



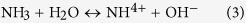



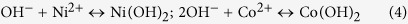


Ni(OH)_6_ and Co(OH)_6_ octahedra nuclei were self-assembled to form the infinite 2D sheets composed of metal cations occupy the centre of octahedra’s edge and hydroxide ions at vertexes. These 2D sheets further extended and form the Ni_50_Co_50_-LDH nanosheets. The nanosheets were washed with ethanol and water. It is expected that H_2_O molecules and NO_3_^−^ ions will be retained within the interlayer space of LDH through hydrogen bond. Importantly, 10 gram scale of Ni_50_Co_50_-LDH can be readily prepared by this simple synthetic method (Fig. S1, SI), which holds great promise for mass production.

**Figure 2:**
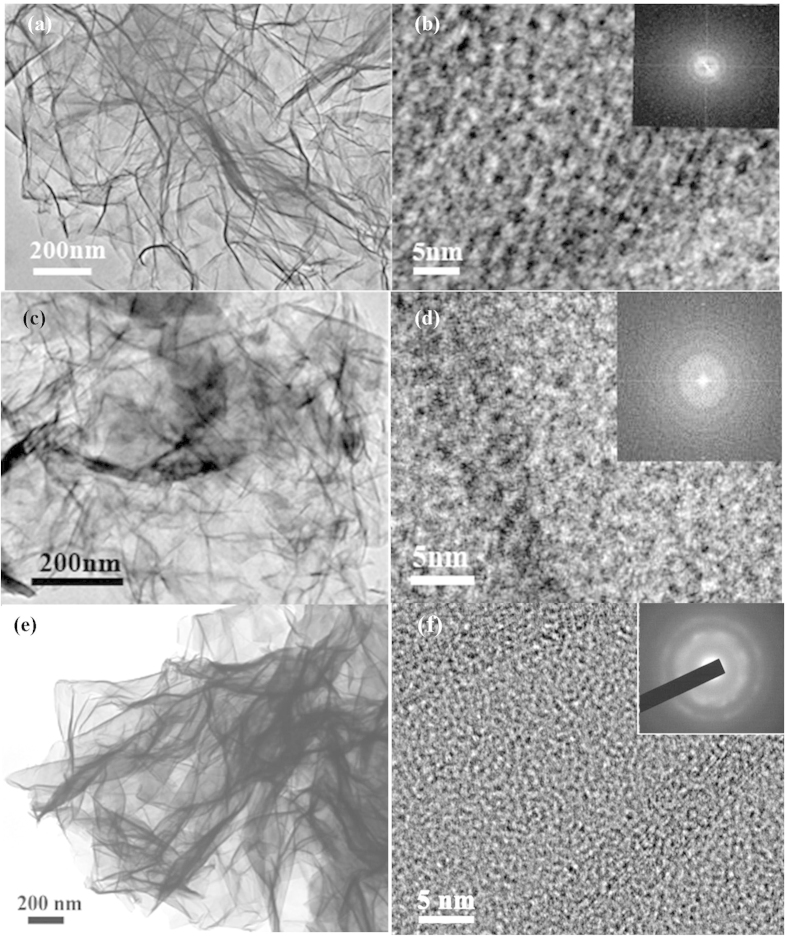
TEM, HRTEM images and SAED patterns of as-prepared samples. *α*-Ni(OH)_2_ (**a**,**b**), *α*-Co(OH)_2_ (**c**,**d**), Ni_50_Co_50_-LDH(**e**,**f**).

The morphology of as-prepared samples was characterized by SEM and TEM techniques. SEM images in Fig. S2 showed that ultrathin nanosheets were about a thickness of ~16 nm. TEM, HRTEM images and the selected-area electron diffraction (SAED) patterns of *α*-Ni(OH)_2_,*α*-Co(OH)_2_ and Ni_50_Co_50_-LDH were shown in Fig. 2. TEM images confirmed that this method could be used to prepare the ultrathin nanosheets transition metal hydroxides and LDHs (Fig. 2a,c,e). In the HRTEM images, lattice fringes were observed on the nanosheets (Fig. 2b,d,f). At the same time, the SAED patterns collected from the nanosheet also exhibited diffraction rings but vague spots, indicating the crystallinity of these samples is relatively low. In addition, some pore structure was also found, which will be advantageous to the electrolyte diffusion.

**Figure 3:**
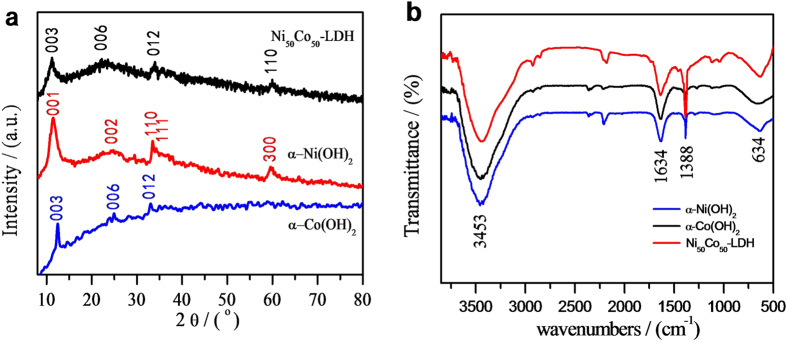
(**a)** XRD patterns and (**b**) FT-IR spectra of the Ni_50_Co_50_-LDH and *α*-Ni(OH)_2_ and *α*-Co(OH)_2_.

The crystal structure of Ni_50_Co_50_-LDH and *α*-Ni(OH)_2_ and *α*-Co(OH)_2_ were further characterized by XRD analysis. As shown in Fig. 3a, the Ni_50_Co_50_-LDH sample exhibited diffraction peaks centered at 11.6°, 23.9°, 34.4° and 60.5° that can be indexed as the (003), (006), (012) and (110) planes of nickel cobalt carbonate hydroxide hydrate (JCPDS 33–0429). The diffraction peaks obtained for Ni(OH)_2_ sample are 12.1°, 24.0°, 33.5°, 35.4° and 59.8°, which can be ascribed to the (001), (002), (110), (111), and (300) planes of layered nickel hydroxide hydrate [*α*-3 Ni(OH)_2_•H_2_O, JCPDS 22–0444]. As-prepared cobalt hydroxide was low-crystalline *α*-hydroxides in good agreement with previous reported results^31^,^32^ showing typical low-crystalline *α*-hydroxides with weak diffraction peaks of (003), (006) and (012) planes in the XRD patterns. The low crystallinity is in accordance with the above-mentioned HRTEM and SAED characterization.

Figure 3b shows the FTIR spectra of Ni_50_Co_50_-LDH, *α*-Ni(OH)_2_ and *α*-Co(OH)_2_ samples. They have similar IR bands. The signal at 3453 cm^−1^ is the O-H stretching band, arising from interlayer water molecules and metal-hydroxyl groups. The band centered at 1634 cm^−1^ can be ascribed to the bending vibration of water. Additionally, the band at 1388 cm^−1^ can be assigned to the vibration of interlayer CO_3_^2−^ and NO_3_^−^ anions. CO_3_^2−^ participated to form the nickel cobalt carbonate hydroxide hydrate with Ni^2+^ and Co^2+^ ions via coordinate bonds while NO_3_^−^ retained in the interlayer of LDH. The broad peak at 634 cm^−1^ can be assigned to the M–O, O–M–O, and M–O–M (M=Co and Ni) vibrations^32^,^33^.

Figure S3a and b show the N_2_ adsorption–desorption isotherms and the corresponding Barret-Joyner-Halenda (BJH) pore size distribution of these samples, respectively. The samples presented a type III curve with H1 hysteresis loop at high relative pressure, indicating the presence of macropores and mesopores. The adsorption isotherms became rapidly saturated at low relative pressure, illustrating the low adsorption volume of metal oxides or LDHs. A platform at *P* / *P*_0_ = 0.20–0.80 originated from the outer superfacial adsorption of nanosheets, contributing the low adsorption volume. In addition, a hysteresis loop at a higher relative pressure (*P* / *P*_0_ = 0.80–0.99) was obtained. This loop resulted from the macroporous adsorption among the overlap gaps of the nanosheets. It was noted that the desorption branch of LDH showed type IV with H2 hysteresis loop, suggesting the existence of mesoporous structure. The BET surface areas of the Ni_50_Co_50_-LDH, *α*-Co(OH)_2_ and *α*-Ni(OH)_2_ were 80, 97 and 119 m^2^g^−1^, respectively, and the average pore size was mainly less than 10 nm. On the other hand, the ion radii (74pm) of Co^2+^ is larger than 72 pm of Ni^2+^, thus with cobalt doping, the interlayer distance was widened and facilitate ion transfer. Nitrogen absorption-desorption measurement indicates that the specific surface of LDHs is increased with the increase of cobalt contents and confirms the presence of mesoporous loop (see Fig. S4).

**Figure 4:**
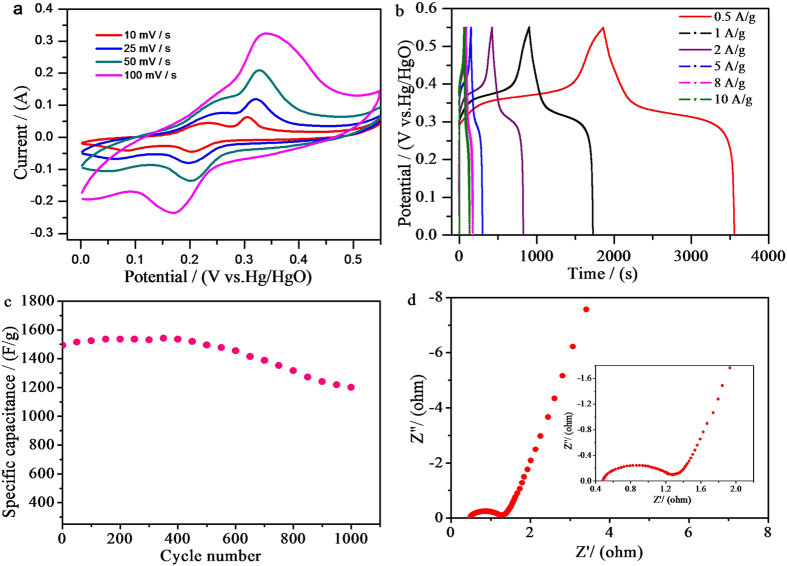
(**a**) CV curves, (**b**) galvanostatic charge/discharge curves, (**c**) cycling stability and (**d**) Nyquist plot of EIS analysis of Ni_50_Co_50_-LDH in 6 mol L^−1^ KOH electrolyte.

Electrochemical measurements were carried out to study the charge storage performance of the Ni_50_Co_50_-LDH samples in 6 mol L^−1^ KOH electrolyte. Figure 4a shows the CV curves for the LDH electrode at different scan rates. A set of distinct redox peaks were observed between 0.1 V and 0.5 V vs. Hg/HgO, which are consistent with the capacitive behavior reported for Ni(OH)_2_ and Co(OH)_2_^34^,^35^. The current intensity increased almost linearly with the increase of scan rate, implying excellent reversibility and rapid charge-discharge response^36^,^37^.

**Figure 5:**
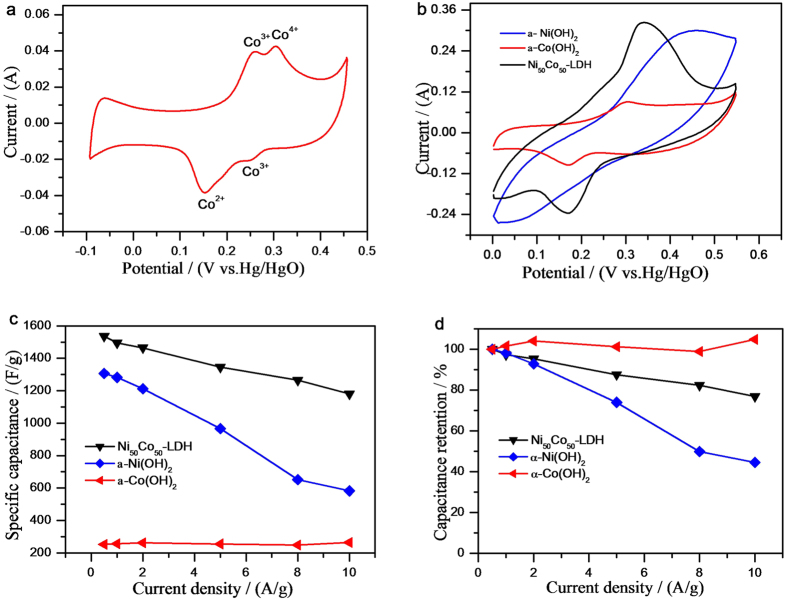
CV curve of (**a**) α-Co(OH)_2_ at 10 mV s^−1^ and (**b**) the Ni_50_Co_50_-LDH, *α*-Ni(OH)_2_ and *α*-Co(OH)_2_ at 100 mV s^−1^. The (**c**) compared specific capacitance and (**d**) the capacitance retention for Ni_50_Co_50_-LDH, α-Co(OH)_2_ and α-Ni(OH)_2_.

The mechanisms of electric energy storage for pseudo-capacitor are proposed as follows (Eq. 5, 6, 7). The pseudo-capacitance of LDH is attributed from both *α*-Co(OH)_2_ and *α*-Ni(OH)_2_. Redox reactions of *α*-Co(OH)_2_ contain two steps as shown in Fig. 5a. The electrons were transported among Co^2+^, Co^3+^ and, Co^4+^ ions with the protons transfer (Eq. 5 and 6). Equation 7 illustrates the charge/discharge mechanism of Ni(OH)_2_.


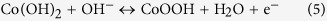



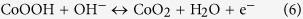



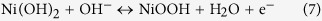


Figure 4b shows the galvanostatic charge-discharge curves of the LDH electrode at different current densities. As a typical battery material, the LDH showed almost symmetrical charge and discharge curves, indicating fast and good electrochemical reversibility. The specific capacitance of the LDH achieved excellent initial specific capactance of 1537 F g^−1^ at 0.5 A g^−1^ and 1181 F g^−1^ even at current density as high as 10 A g^−1^. A 1000-cycle stability curve collected at 2 A g^−1^ is shown in Fig. 4c. The initial specific capacitance was 1494 F g^−1^, the value slowly increased to a maximal value of 1542 around 400^th^ cycle, which was attributed to the activation of Ni-based electrode materials^38^. The retention of the specific capacitance was 80.3% after 1000 cycles. Electrochemical impedance spectroscopy (EIS) was carried out to evaluate the diffusion of electrolyte ions to porous structure and charge transfer at the interface of LDH (Fig. 4d). The impedance plots exhibited two distinct parts including a semicircle in the high-frequency region and a sloped line in the low-frequency region. The charge transfer resistance (*R*_ct_) was estimated to be ~0.8 Ω from the semicircle diameter at the high-frequency. The small R_ct_ could be attributed to the ultrathin nanosheets morphology, which allows efficient charge transfer between the electrolyte and LDH. In addition, the solution resistance (*R*_s_) was estimated to be ~0.48 Ω from the left intersection point of the semi-circle and Z’-axis. The low *R*_ct_ and *R*_s_ as well as high specific capacitance support that the Ni_50_Co_50_-LDH is an excellent capacitive electrode material for ultracapacitors.

The specific capacitance and capacitance retention were measured for Ni_50_Co_50_-LDH, *α*-Co(OH)_2_ and *α*-Ni(OH)_2_ using galvanostatic charge/discharge. As shown in Fig. 5c,d, the specific capacitance of LDH is substantially larger than that of *α*-Ni(OH)_2_ and *α*-Co(OH)_2_ (Fig. 5c). The capacitance of *α*-Ni(OH)_2_ rapidly decreased with the increase of current density. However, the capacitance retention of *α*-Co(OH)_2_ at 10 A g^−1^ is lightly over that at 0.5 A g^−1^ (see Fig. 5d). Though the specific capacitance of *α*-Co(OH)_2_ is much less than that of pure *α*-Ni(OH)_2_, 50% cobalt-doped *α*-Ni(OH)_2_ (Ni_50_Co_50_-LDH) visibly exhibits excellent electrochemical performance, involving superior specific capacitance and the capacitance retention to pure *α*-Ni(OH)_2_. This is also confirmed by the other reseaches. Lang *et al.* obtained Ni_44_Co_56_ oxide nanoflakes with a maximum specific capacitance of 1227 F g^−1^ at 0.625 A g^−1^ based on 0.4 V operating potential^39^. When the atom ratio of nickel and cobalt is close to 1:1, these kinds of materials exhibit the superior electrical conductivity^25^. Cobalt was introduced in LDH to improve the conductivity of electrode materials^25^ and raise the oxygen overpotential advantageous to widening potential window^18^. Co^2+^ were oxidized to form the conductive CoOOH in discharge process, resulting in the increase of conductivity of electrode materials^26^. Due to the cobalt introduced to participate in the electrochemical redox reaction, good conductivity improves the charge transfer and low *R*_ct_ and *R*_s_ are helpful with Faradic reaction, resulting in Ni_50_Co_50_-LDH presents high performance in electrochemical energy storage than nickel hydroxide. The comparable CV curves for the LDH and *α*-Ni(OH)_2_ and *α*-Co(OH)_2_ at 100 mV s^−1^ is shown in Fig. 5b. The serious polarization is shown in CV curve of *α*-Ni(OH)_2_. The reversibility of LDH is visibly improved due to cobalt doped, which is helpful with the Columbic efficiency and the materials utilization.

**Figure 6:**
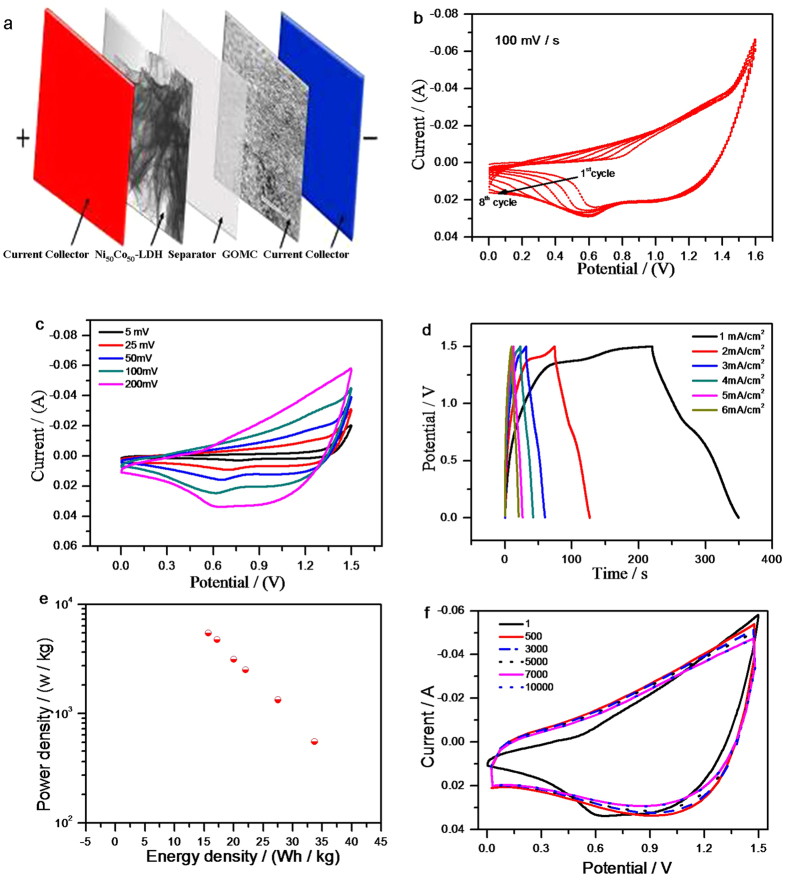
(**a**) Schematic illustration of GOMC//Ni_50_Co_50_-LDH device. (**b**) 1^st^~8^th^ CV curves, (**c**) CV curves at different scan rates. (**d**) Galvanostatic charge/discharge curves. (**e**) Ragone plots of GOMC//Ni_50_Co_50_-LDH. (**f**) CV curves from cycling-stability measurement for GOMC//Ni_50_Co_50_-LDH in 6 mol L^−1^ KOH aqueous electrolyte.

The potential window of Ni_50_Co_50_-LDH is relatively small (~0.55 V), which seriously limit its practical application. In order to enlarge the operating voltage window, we fabricated an asymmetric device using GOMC as negative electrode and Ni_50_Co_50_-LDH as positive electrode (denoted as GOMC//Ni_50_Co_50_-LDH), as shown in Fig. 6a. GOMC is a promising negative electrode material that has long cycling stability and 1.0 V operating potential window in alkaline electrolyte^40^. The CV of GOMC presented a typical rectangular shape in agreement with its electric double-layer capacitive behavior.

The designated asymmetric capacitor has an optimal operating voltage of 1.5 V. When the voltage reached 1.6 V, water splitting occurs and the current drastically increased (Fig. 6b and Fig. S5b). For asymmetric UCs, the charges on anode and cathode should be balanced (*q*+ = *q*−). This can be achieved by manipulating the mass loading of active materials on each electrode. According the following equation, the total charge (*q*) of one electrode stored is depending on the specific capacitance (*C*), the potential window (Δ*E*) and the mass of the electrode (*m*)^41^,^42^.


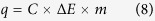


Therefore, the ratio of mass loading of negative and positive electrode materials can be calculated by Eq. 9.


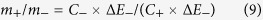


Based on the data of the specific capacitances and potential windows of two electrodes, the optimal mass ratio between GOMC and LDH was 5:1.

Cyclic voltammetry and galvanostatic charge/discharge measurements were collected from the asymmetric UC device. As shown in Fig. 6b, the capacitance of GOMC//Ni_50_Co_50_-LDH asymmetric UC increase gradually due to the activation of nickel hydroxide. Figure 6c shows CV curves measured at different scan rates, the large area of different curves clarified superior performance of this device. Galvanostatic charge/discharge curves were conducted at different current densities (Fig. 6d) to evaluate the capacitance, power density and energy density of asymmetric device. Areal capacitances of 86.3, 70.4, 56.4, 51.2, 44.0 and 40.3 F cm^−2^ were obtained at 1, 2, 3, 4, 5 and 6 mA cm^−2^, which correspond to gravimetric capacitance of 107.8, 88.0, 70.5, 64, 55 and 50.4 F g^−1^, respectively. The Ragone plot for the device is presented in Fig. 6e. The device achieved an excellent energy density of 33.7 Wh kg^−1^ and a high power density of 5.4 kW kg^−1^ (see Formula, SI).

**Figure 7:**
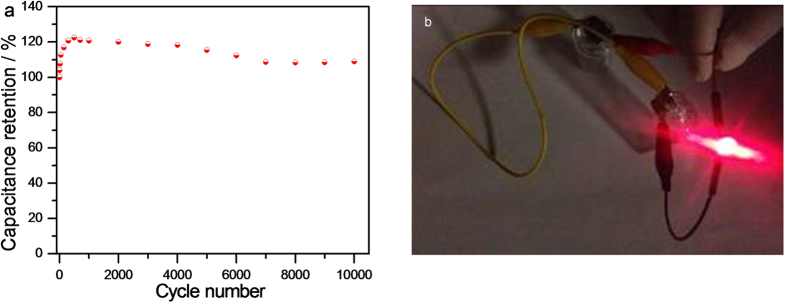
(**a**) Cycling stability for GOMC//Ni_50_Co_50_-LDH device; (**b**) A red LED (1.5 V) was powered by the device.

Cycling stability is a key factor for evaluating the device performance in practical application. Figure 6f shows the CV curves collected at 200 mV s^−1^ as a function of number of cycles. The capacitance quickly increased in the first 500 cycles, which is in good agreement with the Ni_50_Co_50_-LDH electrode performance measured in 3-electrode system. Thereafter, the capacitance decrease gradually. The specific capacitance retention rate was 109% after 10000 cycles (Fig. 7a). The asymmetric device exhibits high specific energy density and excellent cycling stability. These excellent electrochemical performances could be attributed to: (i) the ultrathin and porous nature of Ni_50_Co_50_-LDH and (ii) fast charge transfer, rapid mass transport and anti-corrosion of GOMC. Moreover, GOMC//Ni_50_Co_50_-LDH device could successfully power a red light-emitting-diode (LED) with a nominated voltage of 1.5 V for over 6 min after charging with current density of 4 mA cm^−2^ (Fig. 7b).

## Discussions

Based on the above analysis, it could be found the ultrathin Ni_50_Co_50_-LDH has been successfully synthesized by the efficient and low cost strategy. The morphology characterizations showed that ultrathin nanosheets were about a thickness of ~16 nm and the electrochemical results reveal that Ni_50_Co_50_-LDH possesses high specific capacitance. The ultrathin porous nanostructure can not only be beneficial for efficient ion and electron transport but also improve specific surface area to increase active sites for the energy storage. In addition, the excellent conductivity of as-prepared material has demonstrated by the EIS testing which may also attribute to the enhanced capacitance.

In summary, we have demonstrated a scalable and environmentally-friendly strategy for large-scale preparation of ultrathin Ni_50_Co_50_-LDH. The Ni_50_Co_50_-LDH exhibited high pseudo-capacitance and kinetic properties to be used as the cathode materials for electrochemical energy storage. Therefore, we have developed the asymmetric capacitor composed of Ni_50_Co_50_-LDH and GOMC, which exhibits wide operating voltage of 1.5 V, excellent stability (109% capacitance retention after 10000 cycles), high energy density (33.7 Wh kg^−1^) and power density (5452 W kg^−1^). We believe this novel strategy can be extended to prepare other ultrathin 2D capacitive materials for charge storage devices.

## Methods

### Preparation of NiCo layered double hydroxides

The NiCo LDH was prepared by the following optimal procedure. 2.5 mmol of Ni(NO_3_)_2_•6H_2_O and 5 mmol of Co(NO_3_)_2_•6H_2_O (Ni:Co = 1:2) were dissolved in a mixture solvent of 37.5 mL ethylene glycol and 15 mL deionized water. Then, 37.5 mmol of urea was added under stirring. The resulting solution was transferred into in a round-bottom flask to be refluxed under vigorous magnetic stirring for 3 h at 90 °C. Then, the precipitates were filtered and washed several times with distilled water and ethanol, and then dried at 60 °C. The as-prepared sample was denoted as Ni_50_Co_50_-LDH. The same experimental procedures were also employed to prepare ultrathin Ni(OH)_2_, Ni_79_Co_21_-LDH, Ni_76_Co_24_-LDH, Ni_64_Co_46_-LDH, Ni_35_Co_65_-LDH and Co(OH)_2_ by changing the ratio of the nickel and cobalt source.

### Characterization

Powder X-ray diffraction measurements were performed by a MSAL-XD2 X-ray diffractometer (Cu *K*α, 36 kV, 20 mA, *λ *= 1.5406 Å). The morphologies of LDH samples were examined by field-emission scanning electron microscope (SEM) (FSEM, ZEISS Ultra 55) and high resolution transmission electron microscope (TEM) (HRTEM, JEOL JEM-2100F) with an accelerating voltage of 200 kV. The FT-IR spectra were collected by a Nicolet 6700 FT-IR spectrometer. Nitrogen sorption isotherms of samples were collected by a Micromeritics TriStar 3000 Analyzer at 77 K. Elemental analysis was performed by the inductively coupled plasma optical emission spectrometer (Perkin Elmer, optima 2000DV), indicating the Ni/Co atom ratio of LDH.

### Electrochemical measurements

Working electrode was fabricated by sandwiching the mixture of active materials (8 mg), carbon black and PTFE (with a mass ratio of 80:15:5) between two pieces of nickel foams. The mass loading of the electrode was measured by the mass difference before and after sandwiching. A nickel foil and an Hg/HgO electrode were used as current collector and reference electrode, respectively. All electrochemical measurements were performed on a CHI660D electrochemical workstation in a standard three electrodes cell at room temperature. Cyclic voltammetry (CV), galvanostatic charge-discharge and electrochemical impedance spectroscopy (EIS) tests were all performed in 6 mol L^−1^ KOH aqueous solution. EIS analysis was performed at the frequency range of 100 kHz ~0.1 Hz with amplitude of 5 mV. Asymmetric capacitors were fabricated by using N-doped graphitic ordered mesoporous carbon (GOMC) as negative electrode and Ni_50_Co_50_-LDH as positive electrode, and their electrochemical performance was measured in 6 mol L^−1^ KOH aqueous solution by a 2-electrode cell system.

## Additional Information

**How to cite this article**: Li, R. *et al.* Large Scale Synthesis of NiCo Layered Double Hydroxides for Superior Asymmetric Electrochemical Capacitor. *Sci. Rep.* **6**, 18737; doi: 10.1038/srep18737 (2016).

## Supplementary Material

Supporting Information
