# Supplementary material for: Large Scale Synthesis of NiCo Layered Double Hydroxides for Superior Asymmetric Electrochemical Capacitor
Source: Sci Rep. 2016 Jan 12;6:18737. doi: 10.1038/srep18737 (PMC4709638; doi:10.1038/srep18737)
Supplement: Supporting Information [file srep18737-s1.doc]

**Supporting Information**

**Large Scale Synthesis of NiCo Layered Double Hydroxides for Superior Asymmetric Electrochemical Capacitor**

*Ruchun Li, Zhaoxia Hu, Xiaofeng Shao, Pengpeng Cheng, Shoushou Li, Wendan Yu, Worong Lin, Dingsheng Yuan**

Department of Chemistry, Jinan University, Guangzhou 510632, PR China

Corresponding authors: D.S. Yuan ([tydsh@jnu.edu.cn](mailto:tydsh@jnu.edu.cn))

**Formula**

The specific capacitance (*C*), energy density (*E*) and power density (*P*) are calculated by the following equations:

*C=I*Δ*t***/**(*m*Δ*V*) (1)

*E=*0.5*C*Δ*V*^2^  (2)

*P=E*/Δ*t*  (3)

where *C* (F/g) is gravimetric capacitance, *E* (Wh/kg) is energy density, *P* (W/kg) is power density, *I* (A) is discharge current, Δ*t* (s) is discharge time, *m* (g) is active materials and Δ*V* (V) is operating potential window.

**Table S1** The specific capacitance of the Ni/Co LDHs calculated from discharge curves at different current densities.

| Sample | 0.5  A/g | 1  A/g | 2  A/g | 5  A/g | 8  A/g | 10  A/g |
| --- | --- | --- | --- | --- | --- | --- |
| Ni_79_Co_21_-LDH | 1515 | 1471 | 1451 | 1391 | 1238 | 1173 |
| Ni_76_Co_24_-LDH | 1451 | 1407 | 1385 | 1318 | 1251 | 1100 |
| Ni_64_Co_46_-LDH | 1525 | 1465 | 1418 | 1300 | 1222 | 1163 |
| Ni_50_Co_50_-LDH | 1537 | 1495 | 1465 | 1345 | 1265 | 1181 |
| Ni_35_Co_65_-LDH | 1430 | 1418 | 1389 | 1281 | 1149 | 1018 |


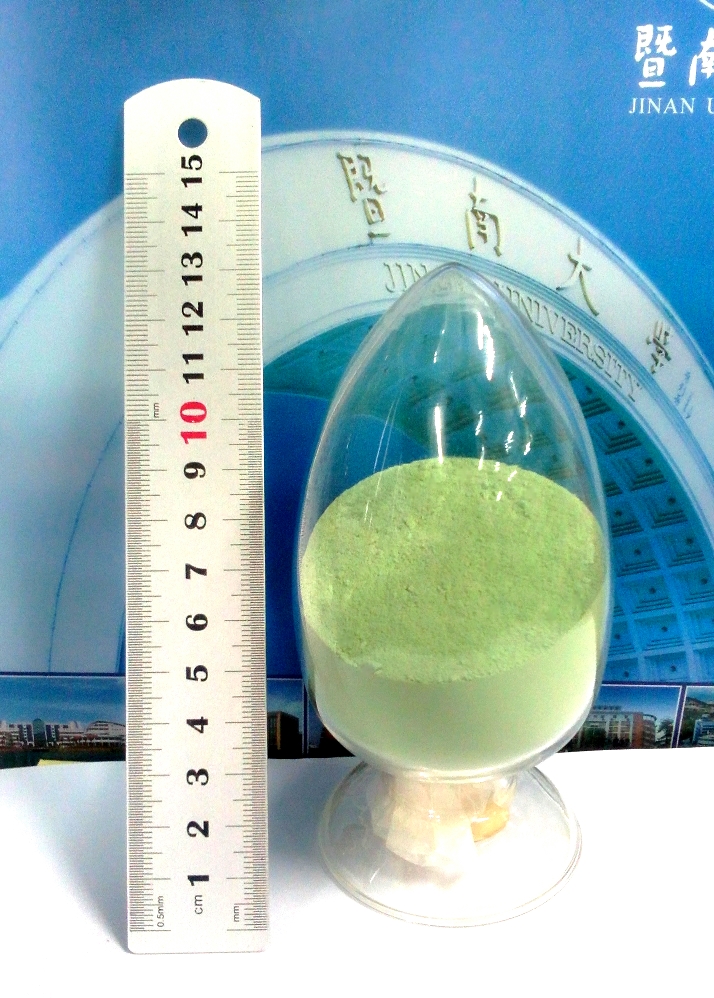


Fig. S1 The photograph of large scale synthesis of NiCo layered double hydroxides. （more than 10g）


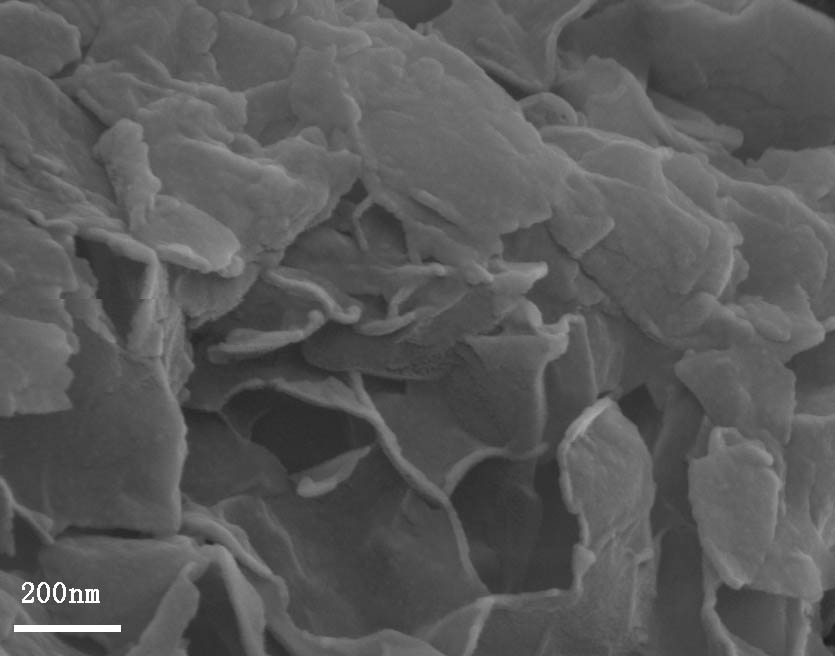


16nm

Fig. S2 The SEM image of Ni_50_Co_50_-LDH.

Fig. S3 (a) Nitrogen adsorption-desorption isotherms and (b) pore size distribution curves of the LDH and *α* -Ni(OH)_2_ and *α* -Co(OH)_2_.

Fig. S4 Nitrogen adsorption-desorption isotherms of the different LDHs.

Fig. S5 (a) The assemble CV curve (a) of GOMC and Ni_50_Co_50_-LDH at 50 mV / s and (b) CV curve of the GOMC//Ni_50_Co_50_-LDH at operating potential window of 1.5V and 1.6V.
